# Rice LecRK5 phosphorylates a UGPase to regulate callose biosynthesis during pollen development

**DOI:** 10.1093/jxb/eraa180

**Published:** 2020-04-09

**Authors:** Bin Wang, Ruiqiu Fang, Jia Zhang, Jingluan Han, Faming Chen, Furong He, Yao-Guang Liu, Letian Chen

**Affiliations:** 1 State Key Laboratory for Conservation and Utilization of Subtropical Agro-Bioresources, South China Agricultural University, Guangzhou, China; 2 Guangdong Provincial Key Laboratory of Protein Function and Regulation in Agricultural Organisms, South China Agricultural University, Guangzhou, China; 3 Guangdong Laboratory for Lingnan Modern Agriculture, Guangzhou, China; 4 College of Life Sciences, South China Agricultural University, Guangzhou, China; 5 Dongyang Institute of Maize Research, Zhejiang Academy of Agricultural Sciences, Dongyang, Zhejiang, China; 6 University of Nottingham, UK

**Keywords:** Callose, lectin receptor-like kinase, *Oryza sativa*, pollen development, rice, UGPase

## Abstract

The temporary callose layer surrounding the tetrads of microspores is critical for male gametophyte development in flowering plants, as abnormal callose deposition can lead to microspore abortion. A sophisticated signaling network regulates callose biosynthesis but these pathways are poorly understood. In this study, we characterized a rice male-sterile mutant, *oslecrk5*, which showed defective callose deposition during meiosis. *OsLecRK5* encodes a plasma membrane**-**localized lectin receptor-like kinase, which can form a dimer with itself. Moreover, normal anther development requires the K-phosphorylation site (a conserved residue at the ATP-binding site) of OsLecRK5. *In vitro* assay showed that OsLecRK5 phosphorylates the callose synthesis enzyme UGP1, enhancing callose biosynthesis during anther development. Together, our results demonstrate that plasma membrane-localized OsLecRK5 phosphorylates UGP1 and promotes its activity in callose biosynthesis in rice. This is the first evidence that a receptor-like kinase positively regulates callose biosynthesis.

## Teaser

Plasma membrane-localized OsLecRK5 enhances the activity of UGP1 by phosphorylation for callose biosynthesis during microsporogenesis in rice.

## Introduction

In pollen development, callose, a polysaccharide of β-1,3-glucan with β-1,6-branches (Chen and Kim, 2009), is biosynthesized and deposited outside the plasma membrane (PM) during meiosis, forming a temporary layer that separates pollen mother cells (PMCs) within an anther locule. The callose wall degenerates at the late tetrad stage, releasing microspores, which are surrounded by the developing exine (Dong *et al.*, 2005; Ariizumi and Toriyama, 2011). Abnormal callose accumulation and degradation during anther development may cause abortive male gametophyte development (Ariizumi and Toriyama, 2011; Shi *et al.*, 2015). In addition, callose deposition plays important roles in the responses to biotic and abiotic stresses such as pathogen infection, wounding, and aluminum toxicity in plants (Sivaguru *et al.*, 2000; Nakashima *et al.*, 2003; Voigt, 2014).

Callose biosynthesis occurs in two steps. UDP-glucose pyrophosphorylase (UGPase) converts glucose-1-phosphate and UTP to UDP-glucose and pyrophosphate; then, glucan synthase-like (GSL) enzymes use UDP-glucose as a substrate to produce callose (Chen *et al.*, 2007). Studies in *Arabidopsis thaliana* and rice (*Oryza sativa*) have demonstrated that disrupting the genes encoding UGPase or GSL alters callose metabolism, causing defective microsporogenesis and, ultimately, male sterility (Enns *et al.*, 2005; Chen *et al.*, 2007, 2009; Huang *et al.*, 2009, 2013; Shi *et al.*, 2015). During tetrad dissolution, a β-1,3-glucanase catalyzes the hydrolysis of β-1,3-glucan to degrade callose; disrupting this β-1,3-glucanase causes male sterility (Wan *et al.*, 2011).

The rice genome contains two UGPase genes (*UGP1* and *UGP2*) and 10 GSL genes (Chen *et al.*, 2007; Shi *et al.*, 2015). *UGP1* affects callose deposition during PMC meiosis and microspore development (Chen *et al.*, 2007). Rice GSL5 is critical for callose biosynthesis during microsporogenesis, as *GSL5* knockout or knockdown causes defective callose deposition on meiocyte cell walls and tetrad cell plates, resulting in male sterility (Shi *et al.*, 2015). How UGPase and callose biosynthesis are regulated during microspore development remains largely unknown.

Receptor-like kinases (RLKs) are a large protein family in plants. RLKs localize to the cell surface, where they perceive extracellular cues and transmit them as intracellular signals (Shiu and Bleecker, 2001). Lectin RLKs (LecRLKs), a major subgroup of RLKs, contain an N-terminal carbohydrate-binding lectin domain and function in diverse roles from plant development to biotic and abiotic stress responses (Navarro-Gochicoa *et al.*, 2003; Wan *et al.*, 2008). The *sgc* (*small, glued-together, and collapsed pollen*) mutant is caused by the mutation of a *LecRLK* gene in Arabidopsis (Wan *et al.*, 2008). LecRLK1 interacts with the N-terminus of AtGSL6, potentially regulating cell plate formation (Dong, 2004). However, these studies did not identify the underlying molecular mechanism.

The rice genome is predicted to contain approximately 173 genes encoding LecRLKs (OsLecRKs) but only a few have been characterized. *OsLecRK1*, *OsLecRK2*, *OsLecRK3*, and *OsLecRK4* form a gene cluster, with the first three genes conferring resistance to the rice predator brown planthopper (Liu *et al.*, 2015*b*). Another rice LecRLK protein, OsLecRK, regulates seed germination and resistance to diseases and insects through actin-depolymerizing factor (Cheng *et al.*, 2013). An important rice blast resistance gene, *Pi-d2*, encodes a LecRLK that confers resistance to *Magnaporthe oryzae* (Chen *et al.*, 2006). Although the functions of LecRLKs have been examined in other contexts, how LecRLKs regulate male sterility has not been elucidated.

In this study, we identified a male-sterile mutant, *oslecrk5*, which is caused by a point mutation in *OsLecRK5*. The callose wall surrounding each tetrad during male reproductive development is defective in the *oslecrk5* mutant. *OsLecRK5* is preferentially expressed in anthers and OsLecRK5 localizes to the PM. We established that a conserved lysine residue, K418, controls OsLecRK5 kinase activity and OsLecRK5 phosphorylates the UGPase UGP1 to increase its activity, thus revealing a mechanism by which this LecRLK controls callose biosynthesis during anther development.

## Materials and methods

### Plant materials and growth conditions

Rice plants were grown in South China Agricultural University’s paddy field. A mapping population was generated from a cross between *oslecrk5* (*japonica*) and Huanghuazhan (HHZ; *indica*). For mapping, eight pairs of insertion/deletion molecular markers were designed based on polymorphisms between the *japonica* and *indica* genomes (see Supplementary Table S1 at *JXB* online).

### Mutant phenotype characterization

Photographs of whole plants, flowers, anthers, and panicles were taken with a Canon digital camera and a dissecting microscope (Olympus SZx10/DP72). Pollen grains were stained with 1% I_2_–KI solution and observed under a microscope (Olympus CX31). Young spikelets with developing anthers were fixed with 3% (w/v) paraformaldehyde and 0.25% glutaraldehyde in 0.1 M phosphate buffer, pH 7.0, and embedded in Epon 812 resin; semithin sections of 2 μm thickness were cut using a Leica RM 2135 microtome, stained with 0.25% toluidine blue, and photographed using a microscope (Zeiss Axiovert 200).

### Callose staining with aniline blue

To observe callose layers, transverse sections of anthers were stained for 10 min at room temperature with 0.01% (w/v) aniline blue in 0.077 M phosphate buffer (pH 8.5; Lu *et al.*, 2014). After being washed with phosphate buffer, sections were visualized under UV light with a confocal laser scanning microscope (Zeiss LSM 7 DUO).

### Plasmid construction and rice transformation

For functional complementation, two binary constructs (driven by the native *OsLecRK5* promoter) expressing the wild-type *OsLecRK5* (*pNP::OsLecRK5*) or *OsLecRK5* fused with a *FLAG* tag (*pNP::OsLecRK5-F*) were prepared. For knockout of *OsLecRK5*, a binary construct (*OsLecRK5*-KO) was designed and prepared using the CRISPR-GE (Xie *et al.*, 2017) and CRISPR/Cas9 (Ma *et al.*, 2015) systems. Mutations of the target site of T_0_ plants were sequenced and analyzed using the DSDecode program (Liu *et al.*, 2015*a*). To analyze the expression pattern of *OsLecRK5*, a construct *pNP::GUS*, in which the β-glucuronidase (*GUS*) reporter gene was driven by the *OsLecRK5* promoter, was prepared for transformation of rice. To test the biological significance of the conserved ATP-binding lysine residue (K418) of OsLecRK5, site-directed mutagenesis using Ω-PCR (Chen *et al.*, 2013) was used to create *pNP::OsLecRK5*^*K418E*^ from *pNP::OsLecRK5*. The *pNP::OsLecRK5*, *pNP::OsLecRK5-F*, and *pNP::OsLecRK5*^*K418E*^ constructs were transferred into induced seed calli from the segregant progeny of heterozygous mutant plants (*OsLecRK5/oslecrk5*); the *OsLecRK5*-KO and *pNP::GUS* constructs were transferred into the *japonica* variety Zhonghua 11 (ZH11) by *Agrobacterium*-mediated transformation.

### Quantitative reverse transcription–PCR analysis

For *OsLecRK5* expression analysis, total RNA from rice organs (roots, culms, leaves, and anthers at different stages) was isolated using Trizol reagent (Thermo Fisher Scientific). Total RNA was used to synthesize cDNA from each sample using M-MLV Reverse Transcriptase (Promega) according to the manufacturer’s instructions. Quantitative reverse transcription–PCR (qRT–PCR) was conducted using the iQSYBR Green Supermix Detection System (Bio-Rad) with three biological repeats. The rice gene *OsActin1* was used as an internal control to normalize target gene expression. Relative expression levels were measured using the 2^(−ΔΔCt)^ method. Gene-specific primers used for qRT–PCR are listed in Supplementary Table S1.

### Subcellular localization and bimolecular fluorescence complementation analysis

To produce the *OsLecRK5-GFP* construct, the coding region of *OsLecRK5* was cloned into the vector pD1-N-GFP (Q. L. Zhu, Y-G. Liu, unpublished data), which carries a *P*_*35S*_*:GFP* cassette. The cytomembrane marker RAC3-mCherry was transiently coexpressed in rice leaf sheath protoplasts by polyethylene glycol-mediated transformation (Chen *et al.*, 2010). To prepare the bimolecular fluorescence complementation (BiFC) constructs, *OsLecRK5*, *UGP1*, and *GSL5* cDNA coding sequences were each cloned into BiFC vectors. Empty vectors and fusion proteins were transiently expressed in *Nicotiana benthamiana* mesophyll cells, and fluorescence images were obtained using a microscope (Zeiss Axiovert 200).

### Pull-down assays

Coding sequences for the OsLecRK5 lectin and kinase domains were cloned into the pMAL-c5X and pET-32a vectors, respectively. Full-length *UGP1* was cloned into the pET-32a vector. Proteins were expressed in *Escherichia coli* BL21 (DE3). TALON® Metal Affinity Resin (TaKaRa Bio) containing 1 μg of maltose binding protein (MBP)-Lectin or MBP was incubated with 1 μg His or His-Lectin in phosphate-buffered saline (PBS). Amylose resin containing 1 μg of MBP-LecRK5-KD or MBP was incubated with 1 μg His or His-UGP1 in PBS. The mixtures were rotated at 4 °C for 8 h. After washing, 20 μl samples were loaded on to a 12% SDS-PAGE gel, and proteins were detected by western blot analysis using anti-MBP or anti-His antibodies (TransGen Biotech) and visualized with Enhanced Chemiluminescence Reagent (Bio-Rad).

### *In vitro* kinase assay

*UGP1* and *LecRK5-KD* were cloned into pET30a and pMAL-C5X vectors, respectively. Proteins were expressed in *E. coli* BL21 (DE3). For *in vitro* kinase assays, His-UGP1 proteins were incubated with MBP or MBP-LecRK5-KD at room temperature for 45 min in a 24 μl reaction mixture containing 20 mM Tris–HCl (pH 7.5), 10 mM MgCl_2_, 5 mM dithiothreitol, and 100 μM ATP. Reactions were terminated by adding 6 μl of 5× SDS loading buffer, and samples were separated by 12% SDS-PAGE (Wang *et al.*, 2018). UGP1 phosphorylation was detected with anti-pY/T/S antibodies (Thermo Fisher Scientific).

### UGPase enzyme activity assay

Recombinant proteins were expressed in *E. coli* BL21 (DE3). Reaction mixtures (1 ml, pH 8.5) contained 80 nM glycylglycine, 1 nM UDP-glucose, 5 nM MgCl_2_, 1 unit each of phosphoglucose mutase and glucose-6-phosphate dehydrogenase, 20 nM Cys, 0.02 nM glucose-1,6-diphosphate, 0.6 nM NADP, and 1 μg of recombinant proteins (Chen *et al.*, 2007). The reactions were initiated by adding 2.5 nM inorganic pyrophosphate. NADPH formation (at 340 nm) was recorded continuously at 30 °C.

## Results

### Identification and characterization of the rice male-sterile mutant *oslecrk5*

**Fig. 1.**
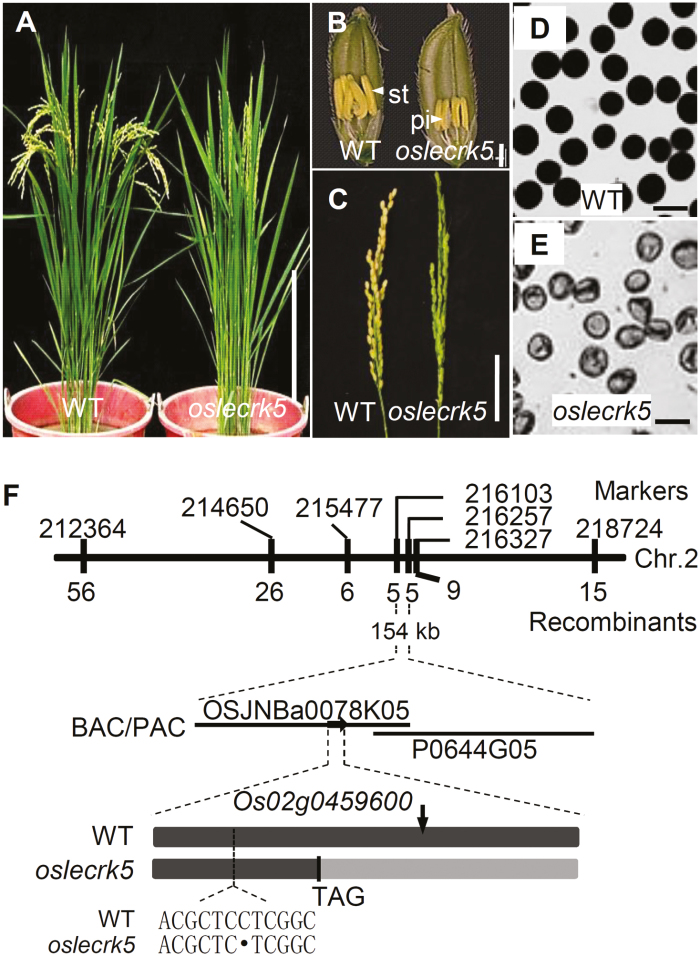
Phenotypic analyses and map-based cloning of *oslecrk5*. (A) Wild-type (WT) and *oslecrk5* plants after heading. Bar=30 cm. (B) WT (left) and *oslecrk5* (right) flowers. st, Stamen; pi, pistil. Bar=1 mm. (C) WT (left) and *oslecrk5* (right) panicles. Bar=4 cm. (D, E) I_2_–KI-stained stage 12 pollen grains from WT (D) and *oslecrk5* (E) anthers. Bars=50 µm. (F) Fine mapping of *OsLecRK5* on chromosome 2. Molecular marker names and positions are indicated. OSJNBa0078K05 and P0644G05 are genomic DNA accession numbers. The *OsLecRK5* locus was mapped to a 154 kb region between two molecular markers (216103 and 216257). The *Os02g0459600* coding sequence (intron free) in *oslecrk5* had a deleted C in the coding sequence at the +144 bp position and formed an mRNA with a premature stop codon. The arrow indicates the target site for CRISPR/Cas9 editing (see Fig. 2C).

We identified a new male-sterile mutant from our rice mutant library (generated by ^60^Co γ-ray irradiation of the *japonica* cultivar 02428; Ji *et al.*, 2013; Niu *et al.*, 2013; Zhou *et al.*, 2017). We named this mutant *oslecrk5* because subsequent analysis showed that its sterility is caused by a mutation in the rice LecRLK gene *OsLecRK5* (see below). *oslecrk5* mutants had normally developing vegetative tissues and female reproductive organs, but they produced pale yellow anthers and shrunken pollen grains (Fig. 1A–E). Genetic analysis showed that all F_1_ plants derived from crossing *oslecrk5* and the *indica* rice variety HHZ displayed a wild-type phenotype. F_2_ plants segregated wild-type and mutant plants in a 146:47 ratio, which is equivalent to a 3:1 ratio (*χ*^*2*^=0.043, *P*>0.05), consistent with a single recessive mutation determining the male sterility of *oslecrk5*.

To identify the gene responsible for the *oslecrk5* mutant phenotype, we used 10 193 F_3_ individuals derived from the cross between the mutant and HHZ to map the target gene within a 154 kb region on chromosome 2 (Fig. 1F). A bioinformatic analysis predicted 21 candidate genes in this region; we amplified and sequenced the candidates that were expressed in anthers. We found that the 144th nucleotide (C) was deleted from the coding region of *LOC_Os02g26160* (http://www.gramene.org/, or *Os02g0459600* by http://rapdb.dna.affrc.go.jp/), resulting in a frame shift (Fig. 1F).

We determined the structure of *Os02g0459600* by employing Rapid Amplification of cDNA Ends (RACE) to compare the genomic region (http://www.gramene.org) with the full-length cDNA and found that the *Os02g0459600* transcript contained a 2088 bp coding sequence. *Os02g0459600* is predicted to encode a LecRLK. Based on the number of previously reported rice *LecRK* genes, we designated *Os02g0459600* as *OsLecRK5*. The predicted OsLecRK5 protein sequence contains 695 amino acids, including an N-terminal lectin domain (amino acids 44–273), one transmembrane region (amino acids 332–354), and a kinase domain (amino acids 359–654; see Supplementary Fig. S1A).

**Fig. 2.**
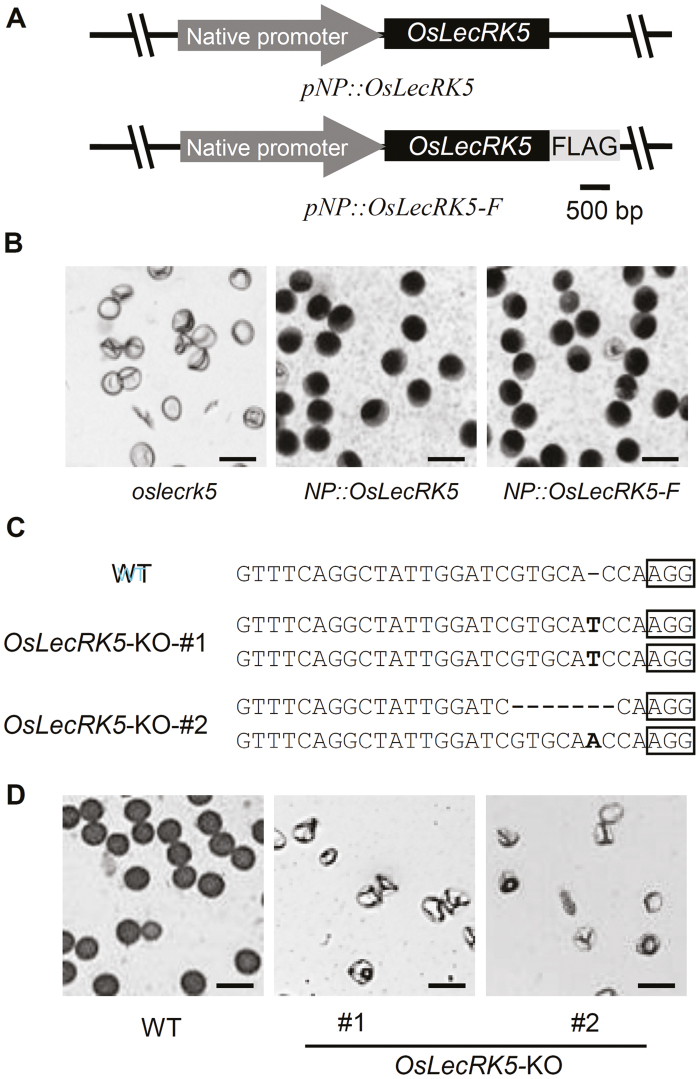
Phenotypes of *OsLecRK5* complemented and knockout plants. (A) Diagrams of *OsLecRK5gDNA* (*pNP::OsLecRK5*) and *OsLecRK5cDNA-FLAG* (*pNP::OsLecRK5-F*) constructs. The native promoter consisted of 3 kb of the *OsLecRK5* 5′ upstream sequence. (B) I_2_–KI-stained stage 12 pollen grains from anthers of *oslecrk5* (left) and OsLecRK5-complemented plants (center and right), showing that expression of *OsLecRK5* in *oslecrk5* recovered pollen deformities. Bars=50 μm. (C) Sequencing of two *OsLecRK5*-KO knockout lines (T_0_) created by CRISPR-Cas9 editing, showing homozygous (#1) and biallelic (#2) mutations. The PAM (protospacer adjacent motif) is boxed. WT, wild type. (D) I_2_-KI-stained stage 12 pollen grains from WT (left) and *OsLecRK5*-KO (center and right), showing that both knockout lines produced deformed pollen. Bars=50 μm.

To confirm the function of OsLecRK5 in pollen development, the coding sequences of *OsLecRK5* (*pNP::OsLecRK5*) or *OsLecRK5* fused with a *FLAG* tag (*pNP::OsLecRK5-F*) were driven by its native promoter and transformed into heterozygous (*OsLecRK5*/*oslecrk5*) plants (Fig. 2A). Both constructs recovered the male-sterile phenotype (Fig. 2B) and the transgene cosegregated with fertility in the T_1_ generation: segregants with transgenes were fertile, and those lacking the transgene were sterile (Supplementary Table S2). In contrast, disrupting *OsLecRK5* by CRISPR/Cas9 editing in the *japonica* rice variety ZH11 (Fig. 2C) caused male sterility (Fig. 2D), indicating that the male sterility of *oslecrk5* was controlled by a single recessive mutation in *Os02g0459600*.

To understand the relationship between rice OsLecRK5 and its homologs in other plant species, we searched public databases [NCBI (https://www.ncbi.nlm.nih.gov/), TAIR (https://www.arabidopsis.org/), Gramene (http://www.gramene.org/), and Ensembl (https://www.ensembl.org/)], querying with the full-length OsLecRK5 sequence. We used the amino acid sequences of 24 reported LecRLKs from *Arabidopsis thaliana*, *Dasypyrum villosum*, *Medicago truncatula*, *Nicotiana attenuata*, *Nicotiana benthamiana*, *Oryza sativa*, *Populus nigra*, and *Pisum sativum* to construct a phylogenetic tree (Supplementary Fig. S1B). The tree grouped OsLecRK5 into a small clade containing SGC, which is necessary for Arabidopsis pollen development (Wan *et al.*, 2008), suggesting that OsLecRK5 may have a conserved function in male gamete development. Rice LecRLKs involved in biotic stress formed a neighboring subgroup (Chen *et al.*, 2006; Cheng *et al.*, 2013; Liu *et al.*, 2015*a*).

### *OsLecRK5* is mainly expressed in anthers and encodes a plasma membrane-localized protein

**Fig. 3.**
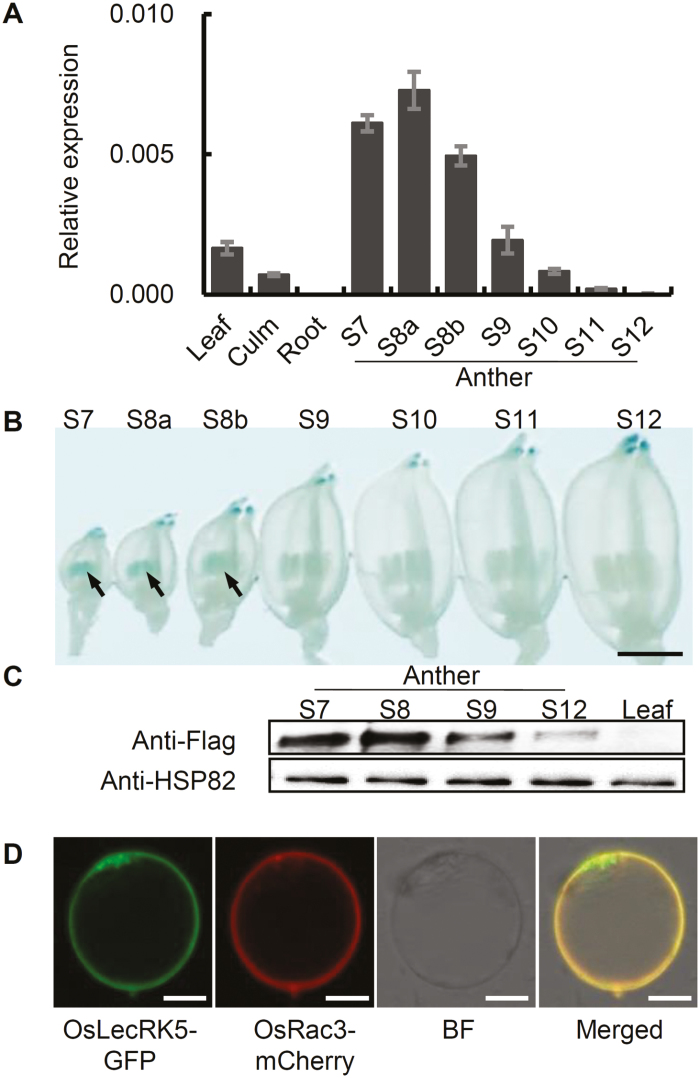
OsLecRK5 expression analysis. (A) *OsLecRK5* expression analysis by qRT–PCR. Relative expression levels indicate the ratios of *OsLecRK5* to *OsActin1* expression. Error bars show SD (*n*=3). (B) Histochemical staining of anthers expressing the GUS reporter gene driven by the *OsLecRK5* promoter. Bar=2 mm. (C) Immunoblot of OsLecRK5 in anthers and leaves. HSP82 is the loading control. (D) OsLecRK5 subcellular localization detected OsLecRK5-GFP predominantly in the plasma membrane (as indicated by the plasma membrane marker Rac3-mCherry). Bars=10 µm.

To investigate *OsLecRK5* expression, we measured its transcript levels in different rice organs using qRT–PCR and found that *OsLecRK5* is expressed in leaves, culms, and anthers, but not in roots (Fig. 3A), with the highest expression in anthers. Interestingly, *OsLecRK5* expression was highest in stage 8 (meiosis stage) anthers and then gradually declined, becoming nearly undetectable by stage 12 (mature pollen stage). These results imply that *OsLecRK5* functions during anther development.

To determine the spatial and temporal patterns of *OsLecRK5* expression *in planta*, we produced plants in which the *GUS* reporter gene was driven by the *OsLecRK5* promoter (*pNP::GUS*) and visualized the GUS signal in flowers and anthers (Fig. 3B). Consistent with the qRT–PCR results, the GUS signal was limited strictly to anthers at the PMC and meiosis stages (stages 7–8), and was very faint at later stages.

To further verify where OsLecRK5 protein accumulates *in planta*, we performed immunoblot analysis of the FLAG-tagged protein OsLecRK5-FLAG expressed under the control of the native *OsLecRK5* promoter (Fig. 3C). Substantiating our previous results, immunoblot analysis showed that OsLecRK5 accumulated mainly in anthers at stage 7−8 (Fig. 3C). Although qRT–PCR detected *OsLecRK5* expression in leaves, our immunoblot analysis barely detected OsLecRK5 in this tissue. Together, these results indicated that although *OsLecRK5* is expressed at low levels in some organs, it is highly expressed in anthers during meiosis, implying that it has a crucial role there.

Like other plant LecRLKs, OsLecRK5 was predicted to contain an N-terminal signal and to localize to the PM (Supplementary Fig. S1A; http://www.cbs.dtu.dk/services/SignalP/). To verify its subcellular localization, we introduced CaMV35S-driven *OsLecRK5-GFP* into rice protoplast cells (Fig. 3D). As expected, the OsLecRK5-GFP signal predominantly colocalized with the PM marker Rac3-mCherry (Chen *et al*, 2010). These results led us to conclude that OsLecRK5 localizes to the PM.

### OsLecRK5 is required for callose deposition during microsporogenesis

To analyze in detail the cellular defects related to the male sterility of *oslecrk5*, we examined transverse anther sections. Anther development in *oslecrk5* was normal prior to PMC meiosis at stage 7 (Supplementary Fig. S2A). Both wild-type and *oslecrk5* anthers consisted of four layers, and PMCs underwent normal meiosis, forming haploid cells by late stage 8 (stage 8b; Supplementary Fig. S2B, C). Between stage 10 and stage 12, the wild-type anther epidermis collapsed and the middle layer, endothecium, and tapetum were mostly degraded. Pollen became vacuolated and underwent normal mitotic divisions to form mature trinucleate pollen grains (Supplementary Fig. S2D−F). By contrast, at stage 12, the *oslecrk5* anther had swollen epidermis and vacuolated pollen grains (Supplementary Fig. S2D−F).

We used scanning electron microscopy imaging to further analyze the cellular defects in *oslecrk5*. At stage 9, the internal surface of the wild-type tapetum contained abundant Ubisch bodies (Supplementary Fig. S2G). In *oslecrk5*, however, the Ubisch bodies were smaller, rounder, and covered by tubular structures (Supplementary Fig. S2G). At stage 12, *oslecrk5* pollen grains had abnormally small apertures compared with wild type (Supplementary Fig. S2H).

Staining with 4′,6-diamidino-2-phenylindole further showed that *oslecrk5* tetrads appeared normal through stage 8b (Supplementary Fig. S3A). However, at stages 9 and 10 (uninucleate microspore stages), *oslecrk5* microspores appeared shrunken (Supplementary Fig. S3B, C). At stages 11–12, each wild-type microspore had completed mitosis and generated trinucleate pollen (Supplementary Fig. S3D, E). By contrast, *oslecrk5* microspores did not undergo proper mitosis and eventually aborted (Supplementary Fig. S3D, E).

**Fig. 4.**
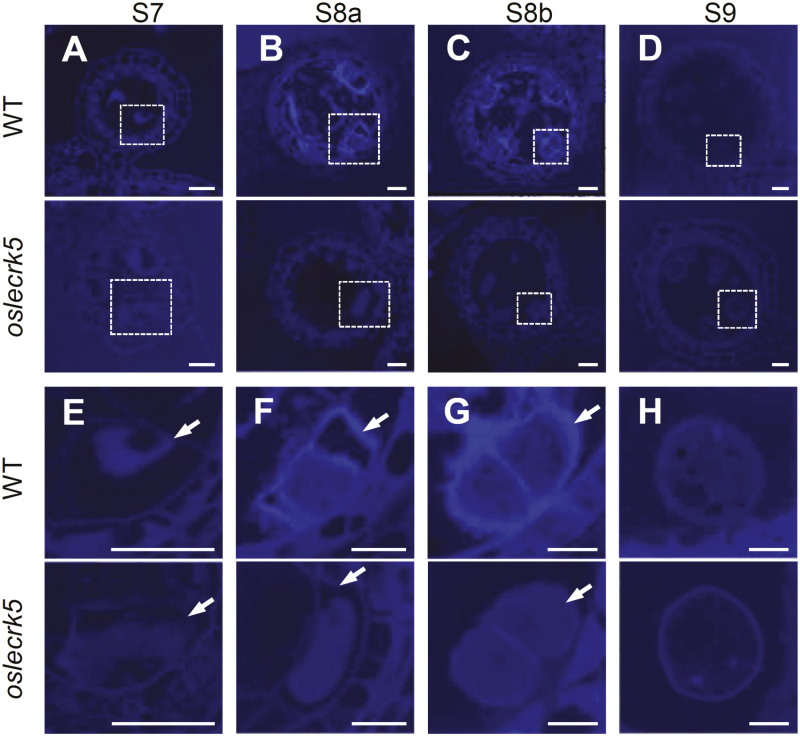
Loss of function of OsLecRK5 causes defective callose deposition during anther development. (A–D) Callose (stained with aniline blue) in wild-type (WT) and *oslecrk5* anther sections from stage 7 (S7) to stage 9 (S9). (E–H) Magnified views of the boxed regions in (A–D) to show details. Arrows indicate callose layers. Bars=10 µm.

The cytological defects indicated that OsLecRK5 may affect anther development prior to the uninucleate microspore stage. We therefore performed aniline blue staining to investigate the early events in callose deposition. Our results showed that wild-type PMCs and tetrads were well formed, with a thick callose wall surrounding each cell during microsporogenesis, and callose was not detectable on released microspores in the wild-type anther (Fig. 4). However, a weak callose staining signal was observed during microsporogenesis in the cell wall of *oslecrk5* anthers (Fig. 4). These results suggested that callose synthesis and/or deposition were impaired in the *oslecrk5* mutant.

### OsLecRK5 interacts with UGP1

**Fig. 5.**
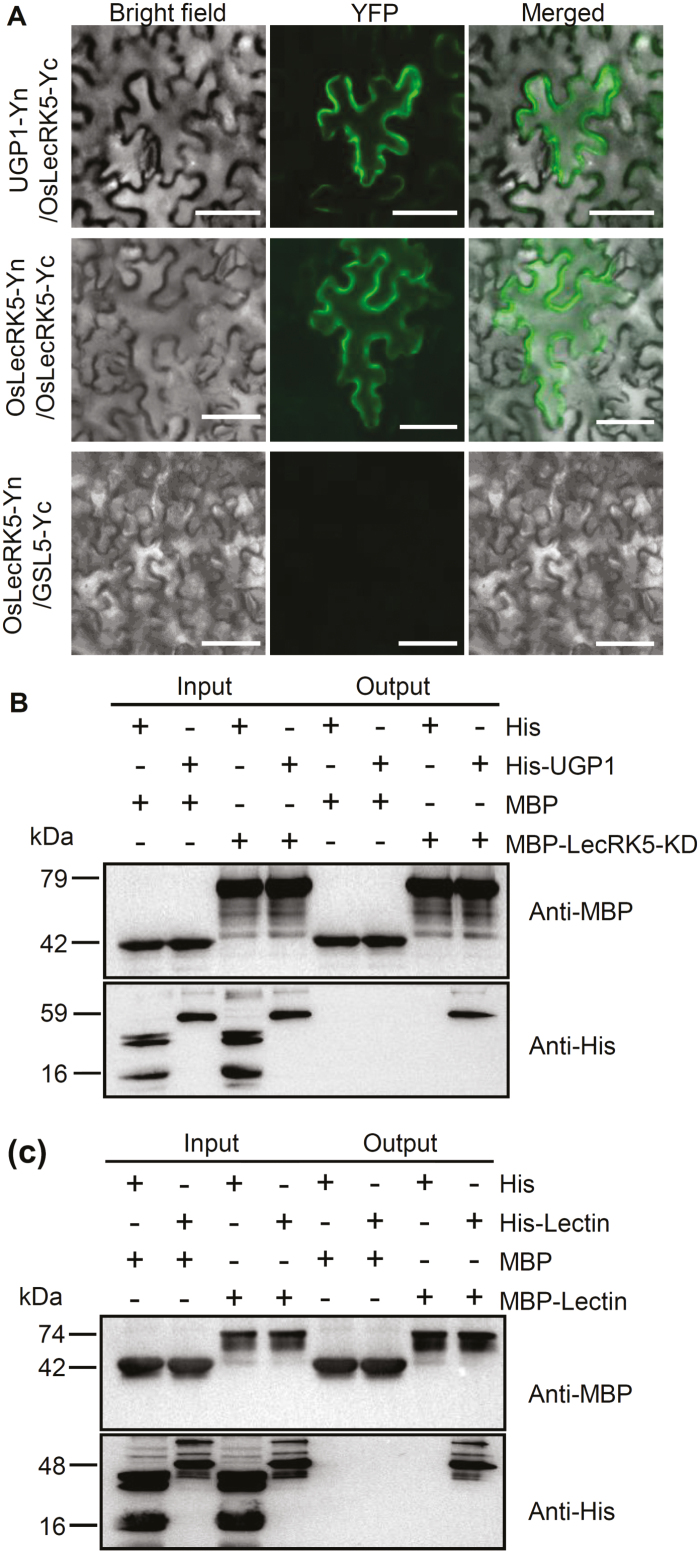
OsLecRK5 homodimerizes and forms a complex with UGP1. (A) Bimolecular fluorescence complementation in tobacco leaves, illustrating the interactions among OsLecRK5, UGP1, and GSL5. Yn, N terminus of yellow fluorescent protein (YFP; amino acids 1–158); Yc, C terminus of YFP (amino acids 155–239). Bars=50 µm. (B) *In vitro* pull-down assay of recombinant MBP-LecRK5-KD (the OsLecRK5 protein kinase domain) and His-UGP1. Maltose binding protein (MBP) alone and His alone served as negative controls. (C) *In vitro* pull-down assay of recombinant MBP-Lectin (the OsLecRK5 lectin domain) and His-Lectin (the OsLecRK5 lectin domain). MBP alone and His alone served as negative controls.

Given that the PM-localized GSL5 and UGP1 proteins are required for callose synthesis in rice anther development (Kimura *et al.*, 1992; Chen *et al.*, 2007; Shi *et al.*, 2015), we hypothesized that OsLecRK5 may directly interact with GSL5 and/or UGP1. Using BiFC assays in tobacco leaves, we found that OsLecRK5 interacts with UGP1 but not with GSL5 *in vivo* (Fig. 5A). We further confirmed this interaction with an *in vitro* pull-down assay using recombinant His-UGP1 and the OsLecRK5 kinase domain tagged with MBP (MBP-LecRK5-KD; Fig. 5B). We found that OsLecRK5 interacted with UGP1, and the OsLecRK5 kinase domain was sufficient to bind UGP1. These results suggested that UGP1 may be a substrate of OsLecRK5 during anther development. Moreover, a BiFC assay showed that OsLecRK5 formed a homodimer and localized on the PM (Fig. 5A). An *in vitro* pull-down assay confirmed the dimerization of OsLecRK5 (Fig. 5C).

### OsLecRK5 activates UGP1 via phosphorylation

**Fig. 6.**
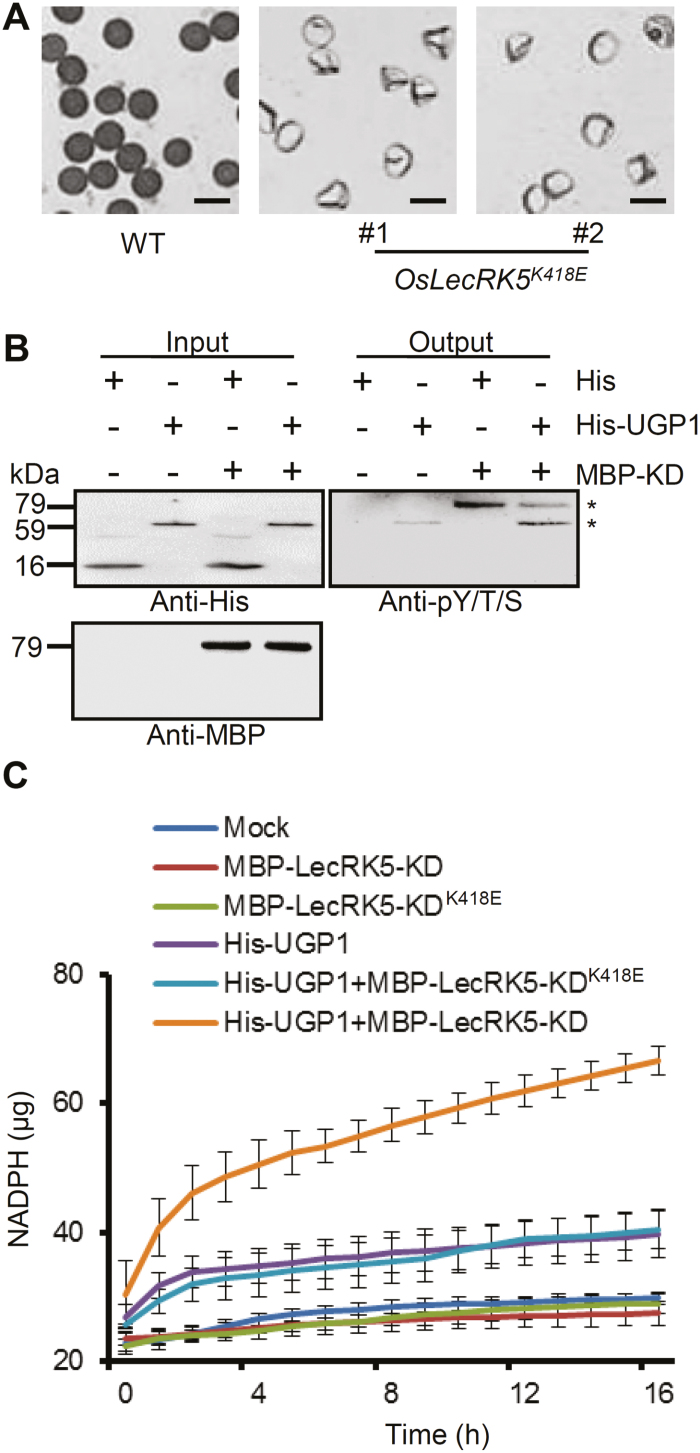
OsLecRK5 phosphorylates UGP1, increasing its activity. (A) I_2_–KI-stained pollen in wild-type (WT, left) and point-mutated transgenic lines (center and right). Bars=50 µm. (B) Phosphorylation assays with OsLecRK5 and UGP1 *in vitro*. Asterisks indicate phosphorylated UGP1 (lower) and phosphorylated OsLecRK5 (upper). (C) UGP1 activity (as µg NADPH) in response to phosphorylation by OsLecRK5.

Sequence analysis showed that the OsLecRK5 kinase domain contains a conserved ATP-binding lysine residue (K418; Supplementary Fig. S1A). To test the biological importance of K418, we introduced a construct carrying a K to E point mutation at this residue (*pNP::OsLecRK5*^*K418E*^) into the *oslecrk5* mutant. Unlike the successful complementation test shown in Fig. 2, OsLecRK5^K418E^ failed to rescue the male sterility of *oslecrk5* in any *OsLecRK5*^*K418E*^ transgenic line (Fig. 6A), suggesting that the kinase function of OsLecRK5 requires K418.

To test whether UGP1 is a substrate of OsLecRK5, we conducted an *in vitro* kinase assay with purified His-UGP1 and MBP-LecRK5-KD. When His-UGP1 and MBP-LecRK5-KD were incubated together with ATP, we detected bands of phosphorylated His-UGP1 and MBP-LecRK5-KD (Fig. 6B), indicating that OsLecRK5 phosphorylates itself and UGP1 *in vitro*.

To understand the relationship between the phosphorylation status of UGP1 and its UGPase activity *in vitro*, we examined UGP1 activity by itself and in the presence of either MBP-LecRK5-KD or the kinase-dead MBP-LecRK5-KD^K418E^. His or MBP-LecRK5-KD alone (negative controls) exhibited no activity, and His-UGP1 alone exhibited weak enzymatic activity (Fig. 6C). However, once MBP-LecRK5-KD was added to His-UGP1, His-UGP1 activity significantly increased. By contrast, adding MBP-LecRK5-KD^K418E^ did not increase His-UGP1 activity. These results clearly indicated that OsLecRK5 phosphorylates UGP1, enhancing its activity and leading to callose biosynthesis.

## Discussion

Pollen development is a complicated and finely tuned process in which callose is first deposited on the PMC surface before meiosis, and forms a temporary cell wall during meiosis (Frankel *et al.*, 1969; Stieglitz, 1977). This callose wall separates meiotic cells and protects them from the environment (Heslop-Harrison and Mackenzie, 1967). After meiosis, the callose wall is degraded, releasing the microspores. Several mutants affected in callose wall metabolism exhibit male sterility (Izhar and Frankel, 1971; Warmke and Overman, 1972; Enns *et al.*, 2005; Chen *et al.*, 2007; Toller *et al*., 2008; Shi *et al.*, 2015). In this work, aniline blue staining demonstrated that initial callose deposition was defective and formation of the callose layer during meiosis was impaired in *oslecrk5* anthers (Fig. 4). The defects of tetrads in *oslecrk5* were not as clear as those in callose biosynthesis-deficient mutants such as *ugp1* and *gsl5* (Chen *et al.*, 2007; Shi *et al.*, 2015), which indicates that *oslecrk5* may still retain a basic level of callose synthesis for the development of tetrads with minor abnormalities, leading to male sterility (Supplementary Fig. S3A).

**Fig. 7.**
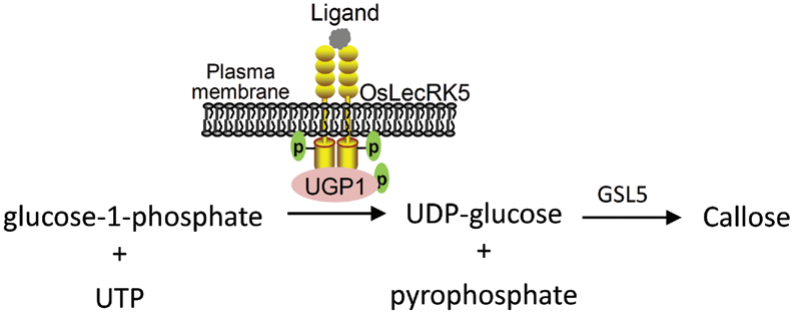
Model of OsLecRK5 regulation in the callose synthesis pathway. OsLecRK5 proteins dimerize in response to a currently unidentified ligand and are activated by autophosphorylation. Active OsLecRK5 then phosphorylates UGP1 to enhance its activity in callose synthesis.

UGPase is a key enzyme in plant carbohydrate metabolism and cell wall biosynthesis (Kleczkowski *et al.*, 2004). A previous study in rice demonstrated that UGP1 knockdown induces abnormal callose deposition during meiosis (Chen *et al.*, 2007), but what regulates UGP1 itself is largely unknown. Our genetic evidence indicated that OsLecRK5 positively regulates callose biosynthesis in rice (Fig. 4). OsLecRK5 interacted with UGP1 *in vivo* and *in vitro* (Fig. 5A, B). We further revealed that OsLecRK5 phosphorylates UGP1 (Fig. 6B), increasing its enzymatic activity (Fig. 6C and Fig. 7). Consistent with our proposed regulatory mechanism, plant UGP1 contains many putative phosphorylation sites (Eimert *et al.*, 1996).

Many LecRLKs are PM-localized receptors (Xin *et al.*, 2009; Huang *et al.*, 2013; Liu *et al.*, 2015*a*). RLKs dimerize or oligomerize in response to external ligands, activating a kinase cascade that amplifies a signal (Shiu and Bleecker, 2001). We found that OsLecRK5 contains an N-terminal signal peptide and localizes to the PM as a homodimer (Supplementary Fig. S1A; Fig. 3D and Fig. 5A). OsLecRK5 is a typical Ser/Thr kinase and has a key lysine residue (K418) at a putative ATP-binding site (Supplementary Fig. S1A). Mutating this lysine (K418E) abolished OsLecRK5 function in pollen development (Fig. 6A). An *in vitro* kinase assay showed that OsLecRK5 can be autophosphorylated (Fig. 6B). These findings imply that the PM-localized LecRLK OsLecRK5 detects a ligand (currently unidentified) and transduces a signal from ligand detection to activate callose biosynthesis during anther development (Fig. 7). Identifying the ligand of OsLecRK5 will be an important goal for future work.

Together, our results reveal that OsLecRK5 phosphorylates UGP1 to activate its activity in callose biosynthesis (Fig. 7). These findings may provide new clues about the regulation of callose biosynthesis related to other stresses and developmental processes.

## Supplementary data

Supplementary data are available at *JXB* online.

Fig. S1. OsLecRK5 sequence analysis.

Fig. S2. Aberrant anther development in *oslecrk5*.

Fig. S3. Microspore development in wild type and *oslecrk5*.

Table S1. Primers used in this study.

Table S2. Genetic analysis of T_1_*OsLecRK5* transgenic plants.

eraa180_suppl_Supplementary_Figures_S1-S3_Table_S1-S2
