# Supplementary material for: Rice LecRK5 phosphorylates a UGPase to regulate callose biosynthesis during pollen development
Source: J Exp Bot. 2020 Apr 9;71(14):4033–41. doi: 10.1093/jxb/eraa180 (PMC7475243; doi:10.1093/jxb/eraa180)
Supplement: eraa180_suppl_Supplementary_Figures_S1-S3_Table_S1-S2 [file eraa180_suppl_supplementary_figures_s1-s3_table_s1-s2.pdf]

The following Supporting Information is available for this article:

Fig. S1 OsLecRK5 sequence analysis.

Fig. S2 Aberrant anther development in *oslecrk5*.

Fig. S3 Microspore development in wild type and *oslecrk5*.

Table S1 Primers used in this study.

Table S2 Genetic analysis of T_1 *OsLecRK5* transgenic plants.

A

```
                signal peptide                          lectin domain
  1 MPPRCRRLPL LFILLLAVRP LSAAAASSIA AAPASSYRRI SWASNLTLLG SASLLPGAAG
 61 VALTTPSRDG VGAGRALFSE PVRLLLPQDA AASASASRAA TPASFSTRFT FRITPSPTYG
121 DGLAFLLTSS RTFLGASNGF LGLFPSSSAS DEGELRDVST VAVEIDTHLD VALHDPDGNH
181 VALDAGSIFS VASAQPGVDL KAGVPITAWV EYRAPRRRLN VWLSYSPSRR PEKPALSADV
241 DLSGLLRTYM YAGFSASNGN GAALHVVERW TFRTFGFPNS SYAPPPTKYI GPMPPNNQPL
                                                  transmembrane region
301 PPPPSPSPSP PPPSPPPPPH PNHRRRHLFY KVLGGVLGGM VLLGLVVVGS AVLLGRSVRR
               kinase domain                                           *
361 KNQEHAVASE DMGEATLSME VARAATKGFD SGNVIGVGGS GATVYEGVLP SGSRVAVKRF
421 QAIGSCTKAF DSELKAMLNC PHHPNLVPLA GWCRSKDELV LVYEFMPNGN LDSALHTLGG
481 ATLPWEARFR AVYGVASALA YLHDECENRI IHRDVKSSNV MLDAEFNARL GDFGLARTVS
541 HGGLPLTTQP AGTLGYLAPE YVHTGVATER SDVYSFGVLA LEVATGRRPA ERGISVVNWV
601 WTLWGRRRLV DAADRRLQGR FVADEMRRVL LVGLCCVHPD CRKRPGMRRV VSMLDGTAPL
661 ILVPDKMPPV LLQPVPNASS MNSADTANTA FFSCR
```

B

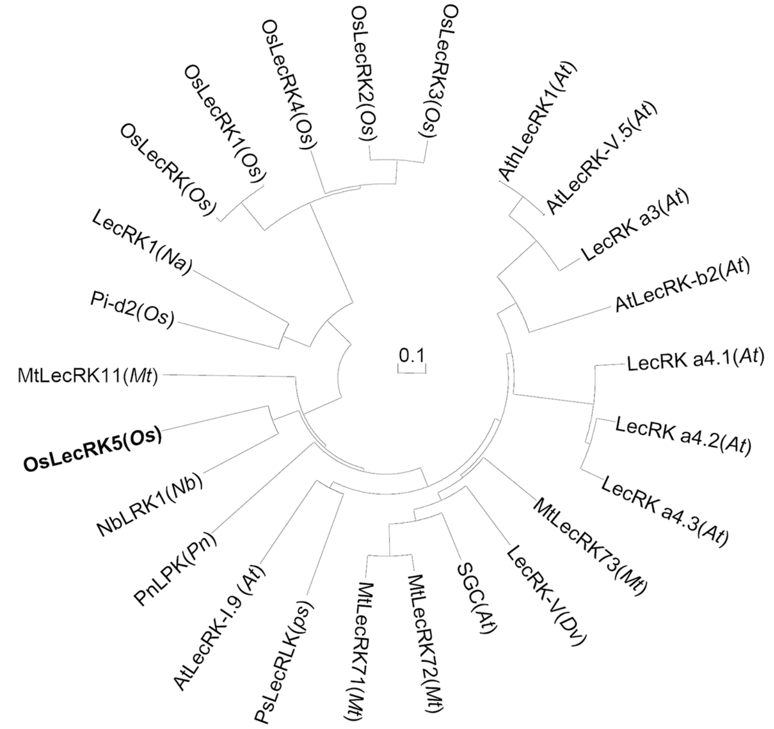

Fig. S1 OsLecRK5 sequence analysis. (A) The putative signal peptide is boxed, the putative lectin domain is underlined, the putative transmembrane region is double underlined, and the putative protein kinase domain is underlined with a dashed line. The asterisk indicates the conserved lysine residue K418. (B) The neighbor-joining phylogenetic tree includes OsLecRK5 (bold) and all characterized lectin receptor-like kinases in plants. *At*, *Arabidopsis thaliana*; *Dv*, *Dasypyrum villosum*; *Mt*, *Medicago truncatula*; *Na*, *Nicotiana attenuata*; *Nb*, *Nicotiana benthamiana*; *Os*, *Oryza sativa*; *Pn*, *Populus nigra*; *Ps*, *Pisum sativum*.

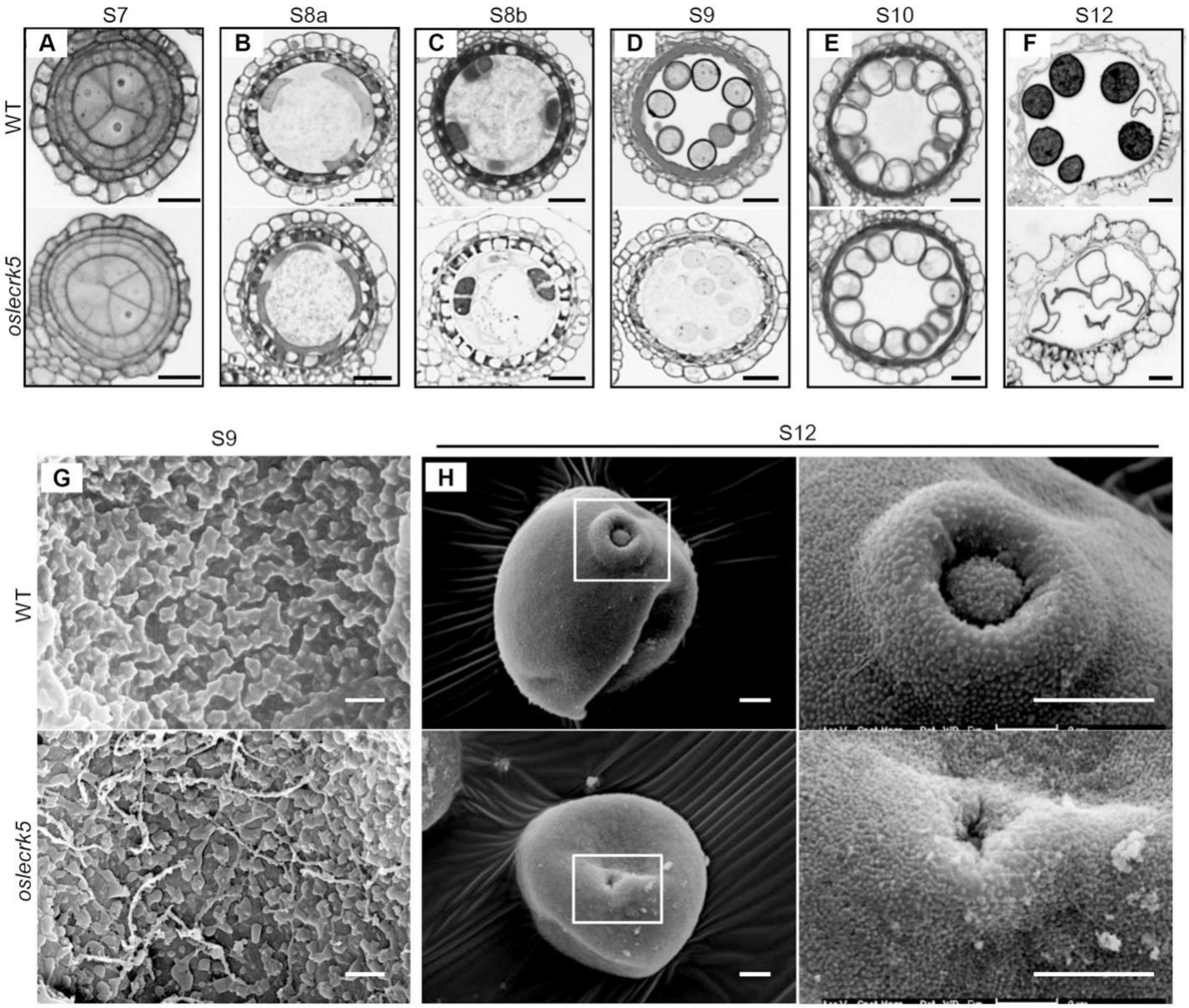

Fig. S2 Aberrant anther development in *oslecrk5*. (A)‒(F), Transverse sections of anther development in wild type (top) and *oslecrk5* (bottom) from stage 7 to stage 12. (G)‒(H), Scanning electron micrographs of the tapetum inner surface (G) at stage 9 and the pollen grain surface (H) at stage 12 in wild type (top) and *oslecrk5* (bottom). Boxed regions in (H) indicate pollen apertures; these regions are magnified on the right. Bars, 15 μm in (A)–(F), 500 nm in (G), and 4 μm in (H).

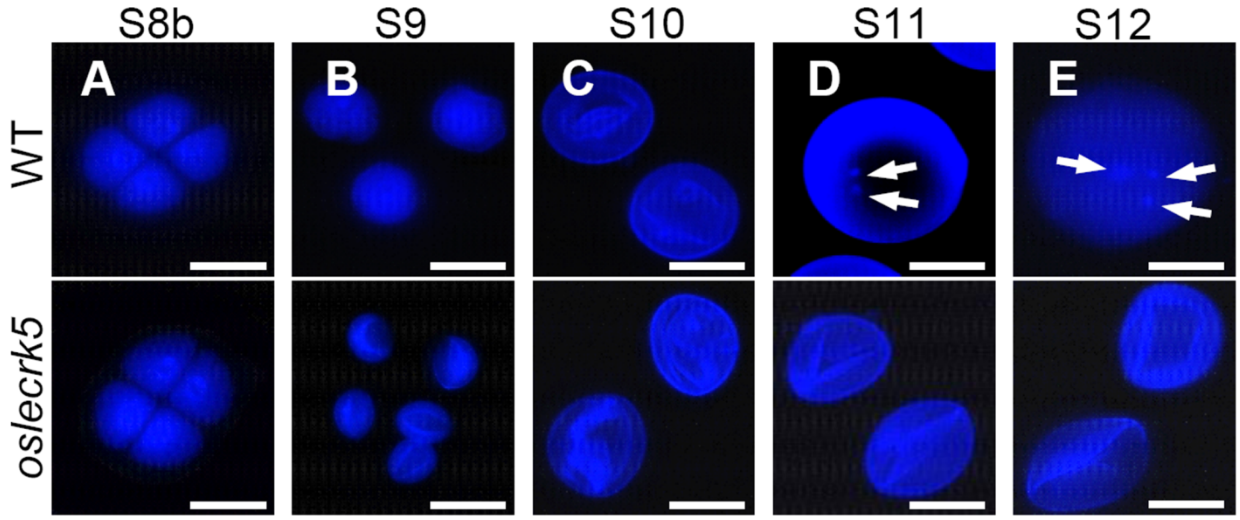

Fig. S3 Microspore development in wild type and *oslecrk5*. DAPI-stained microspores in wild type (top) and *oslecrk5* (bottom) from stage 8b to stage 12. The nuclei are indicated by arrows. Bars, 20 μm in (A)–(E).

Table S1 Primers used in this study.

Primer	Sequence (5′ to 3′)	Purpose
LecRK5-F	GTTGCAGATGAGATGCGAC	qRT-PCR analysis
LecRK5-R	CAGTGCCATATAGGCTGTAG	
212364-F	GCTTGCATAGTGTACGTGCACT	Mapping markers
212364-R	GTGCACTGAGTGCAAATGTGA	
214650-F	GTAAGGGTCCTGTTGGATCT	
214650-R	GAGCATCTCCAACATAGCCT	
215477-F	GCCCGCTATTCTTCTCTCTTAT	
215477-R	GCCCTAGAGCAGGTACAAGT	
216103-F	CCATCCATCGAACAGATGTAGG	
216103-R	CAGTGACAGTAGGTGGAGTG	
216257-F	TATGGCCGCTAGGCACAAAG	
216257-R	TTGAAGGGGGCTTGAAGTGG	
216327-F	GTCAACGTTGAACTGCATGTG	
216327-R	TGCTGCAGCTGTGATGCTAC	
218724-F	CAAGTAGGAGTAGTACTTCTAG	
218724-R	TGACAAGTTAGAAGACATGC	
LecRK5-3R-F1	GAAGGGTAGTCAGTATGCTTGATG	RACE analysis
LecRK5-3R-F2	CACCAGTTCTTCTACAGCCAGTACC	
LecRK5-5R-R1	TGAGGTTGCTCGCCCACGAGATG	
LecRK5-5R-R2	GCAAGGAGGAGGATGAAGAG	
LecRK5-HB-F	CGCGGATCCCCAACGAAGTAAGTACTGAT	Complementation vector
LecRK5-HB-R	CCCAAGCTTCAGGTCATCTTATCAGTGCC	
LecRK5-FLAG-F	TGACATGATTACGAATTCCCAACGAAGTAAGTACTGAT	
LecRK5-FLAG-R	CTGCAGGCATGCAAGCTTGCGACAACTGAAGAATGCAGTA	
LecRK5-KO-F	GTTGGCTATTGGATCGTGCACCA	Gene knockout vector
LecRK5-KO-R	AAACTGGTGCACGATCCAATAGC	
LecRK5-GUS-F	ATATAAGCTTCCAACGAAGTAAGTACTGAT	GUS analysis
LecRK5-GUS-R	ATATCCATGGGGCGATGGCGCTGCTAGGTG	
LecRK5-GFP-F	GCTTCGAATTCTGCAGTCGACATGCCTCCACGCTGTAGGC	Subcellular analysis
LecRK5-GFP-R	TCTAGATCAGGTGGATCCGCGACAACTGAAGAATGCAG	
GSL5-Yc-F	TACAATTACAGGTACCCGGCATTACTCGCATCCATTCTC	BiFC analysis
GSL5-Yc-R	CACCGCCGTCGACTCTAGAAACTGTTCTACAGAGTGTCTG	

Primer	Sequence (5′ to 3′)	Purpose
LecRK5-Yn-F	TACAATTACAGGTACCCGGATGCCTCCACGCTGTAGG	BiFC analysis
LecRK5-Yn-R	TCGCCCTTGCTCACCATACCGCGACAACTGAAGAATGCAG	
LecRK5-Yc-F	TACAATTACAGGTACCCGGATGCCTCCACGCTGTAGG	
LecRK5-Yc-R	CACCGCCGTCGACTCTAGAGCGACAACTGAAGAATGCAG	
UGP1-Yn-R	TCGCCCTTGCTCACCATACCAAGATCCTCCGGACCGTTGA	
UGP1-Yn-F	TACAATTACAGGTACCCGGATGGCGGTCACCGCCGACGTGAA	
Yc-UGP1-F	ATGGACGAGCTGTACAAGGCCATGGCGGTCACCGCCGACGTGAA	
Yc-UGP1-R	CTCTGCAGGTCGACTCTAGATCAAAGATCCTCCGGACCGTTGA	
MBP-lectin-R	GCTTATTTAATTACCTGCAGGACCTTGTAGAACAGATGGC	Pull-down and phosphorylation analysis
MBP-lectin-F	GATCGAGGGAAGGATTTCAGCCGCCGCGTCGAGCATC	
UGP1-His-F	CAAGGCCATGGCTGATATCATGGCGGTCACCGCCGACGT	
UGP1-His-R	GCTCGAGTGCGGCCGCAAAGATCCTCCGGACCGTTGATG	
His-lectin-R	CAAGGCCATGGCTGATATCACCTTGTAGAACAGATGGC	
His-lectin-F	GCTCGAGTGCGGCCGCAGCCGCCGCGTCGAGCATC	
KD-MBP-F	GATCGAGGGAAGGATTTCAGGCCGGTCAGTGCGCCGCAAAAA	
KD-MBP-R	GCTTATTTAATTACCTGCAGGTCAGCGACAACTGAAGAATGC	

Table S2 Genetic analysis of T_1 *OsLecRK5* transgenic plants.

Line	Genotype	No. of plants	Phenotype
NP::OsLecRK5	+ +/+ −	80	Fertile
	− −	17	Sterile
NP::OsLecRK5-FLAG	+ +/+ −	38	Fertile
	− −	11	Sterile

Notes: +, contains the *OsLecRK5* transgene
